# Effects of Hypoxic Preconditioning and Vascular Endothelial Growth Factor on the Survival of Isolated Primary Retinal Ganglion Cells

**DOI:** 10.3390/biom11030391

**Published:** 2021-03-06

**Authors:** Hyoung Won Bae, Wungrak Choi, Ah Reum Hwang, Sang Yeop Lee, Gong Je Seong, Chan Yun Kim

**Affiliations:** Institute of Vision Research, Department of Ophthalmology, Yonsei University College of Medicine, Seoul 03722, Korea; baekwon@yuhs.ac (H.W.B.); wungrakchoi@yuhs.ac (W.C.); ahreum4302@yuhs.ac (A.R.H.); yeopy@yuhs.ac (S.Y.L.); gjseong@yuhs.ac (G.J.S.)

**Keywords:** hypoxic preconditioning, retinal ganglion cell, vascular endothelial growth factor

## Abstract

This study aimed to investigate the effect of hypoxic preconditioning (HPC) on primary retinal ganglion cell (RGC) survival and the associated mechanism, including the role of vascular endothelial growth factor (VEGF). Retinas were separated from the enucleated eyeballs of Sprague–Dawley rats on postnatal days 1–4. RGCs were harvested using an immunopanning-magnetic separation system and maintained for 24 h in a defined medium. Hypoxic damage (0.3% O_2_) was inflicted on the cells using a CO₂ chamber. Anti-VEGF antibody (bevacizumab) was administered to RGCs exposed to hypoxic conditions, and RGC survival rate was compared to that of non-anti-VEGF antibody-treated RGCs. HPC lasting 4 h significantly increased RGC survival rate. In the RGCs exposed to hypoxic conditions for 4 h, VEGF mRNA and protein levels were significantly increased. Treatment with high dose bevacizumab (>1 mg/mL) countered HPC-mediated RGC survival. Protein kinase B and focal adhesion kinase levels were significantly increased in 4-h hypoxia-treated RGCs. HPC showed beneficial effects on primary RGC survival. However, only specifically controlled exposure to hypoxic conditions rendered neuroprotective effects. Strong inhibition of VEGF inhibited HPC-mediated RGC survival. These results indicate that VEGF may play an essential role in promoting cell survival under hypoxic conditions.

## 1. Introduction

Glaucoma is one of the leading causes of blindness worldwide, with the estimated number of patients exceeding 64 million [1,2]. It is a chronic disease that requires lifetime treatment upon diagnosis. Conventionally, treatment begins with medication to lower the intraocular pressure (IOP) to halt the progression of the disease. However, the disease continues to progress in certain patients despite satisfactory decrease in IOP [3,4]. Consequently, studies have searched for additional treatment strategies. Based on the ability of the cells to adapt to stressful conditions, preconditioning-induced, neuroprotection-based approaches to prevent or slow the progressive loss of the retinal ganglion cells (RGCs) have received considerable attention [5].

The retina is one of the most metabolically active tissues and requires large amounts of oxygen—even more than that of the brain. Thus, the proper functioning of the retina is highly dependent on a continuous supply of oxygen [6]. This supply can be impeded by asphyxia (lack of oxygen in the blood stream), hypoxia (reduction in the available oxygen), or ischemia (reduction in blood flow that could lead to hypoxia) [7]. Deficient oxygen supply results in tissue hypoxia, leading to the death of RGCs and subsequent loss of vision in many ocular conditions, including retinal vein occlusion, glaucoma, and diabetic retinopathy [6,8]. However, previous studies have reported that the duration of retinal tolerance to low-oxygen conditions in adult mice can be extended from days to weeks through hypoxic preconditioning (HPC) prior to insult [5]. It is believed that under hypoxic conditions, the neural tissue is capable of inducing protective mechanisms to limit cellular damage and thereby enhance survival.

Several factors have been identified to be critical to the regulation of protective mechanisms during hypoxia, including hypoxia-inducible factor (HIF)-1α, vascular endothelial growth factor (VEGF), and nitric oxide synthase [9]. Studies have shown that the overproduction of these factors is implicated in neuronal death under hypoxic conditions. One of the regulatory factors, VEGF, has been demonstrated to play a central role in the development of various ophthalmic diseases including age-related macular degeneration (AMD), and anti-VEGF antibody has been widely used to treat these diseases [10,11]. Therefore, it is necessary to conduct in-depth studies on the role of VEGF in the protective mechanism of HPC.

The purpose of this study was to investigate the effect of HPC on primary RGC survival and the associated mechanisms, including the functional role of VEGF and anti-VEGF antibody.

## 2. Materials and Methods 

### 2.1. Reagents

For in vitro cell culture, Dulbecco’s Modified Eagle’s Medium/F-12 Nutrient Mixture Ham 1:1 Mixtures (DMEM/F-12) was purchased from HyClone Laboratories (South Logan, UT, USA). Fetal bovine serum was purchased from Gibco (Life Technologies, Grand Island, NY, USA). Poly-L-ornithine solution and laminin solution were procured from Sigma-Aldrich (St. Louis, MO, USA), and penicillin/streptomycin was obtained from Gibco (Life Technologies).

For the preparation of RGCs, antibiotin microbeads were purchased from Miltenyi Biotec (Bergisch Gladbach, Germany). Antimacrophage antibody was obtained from Fitzgerald Industries International (Acton, MA, USA). Anti-immunoglobulin G and anti-Thy1 antibodies were purchased from Southern Biotech (Birmingham, AL, USA) and Bio-Rad (Hercules, CA, USA), respectively. 

For flow cytometry or cell sorting, cells were incubated with the primary antibody Annexin-V purchased from BioVision (Milpitas, CA, USA). Anti-β-actin antibody used for immunoblotting was procured from Santa Cruz Biotechnology (Dallas, TX, USA), and tubulin was obtained from Cell Signaling Technology (Danvers, MA, USA).

### 2.2. Rats

Retinas were separated from the enucleated eyeballs of Sprague–Dawley rats (Orientbio, Seongnam, Republic of Korea) on postnatal days 1–4. The study was approved by the Institutional Animal Care and Use Committee (2019-0150), and the rats were treated according to the Association for Research in Vision and Ophthalmology Statement for the Use of Animals in Ophthalmic and Vision Research.

### 2.3. Primary RGC Harvest and Culture

Primary RGCs were harvested using a previously described immunopanning-magnetic separation method [12]. Briefly, the retinal cell suspension was incubated with antimacrophage antibody and then distributed over an anti-immunoglobulin G antibody-coated petri dish. Nonadherent cells were treated with biotinylated anti-Thy-1.2 antibody and subsequently allowed to interact with antibiotin microbeads. Finally, the magnetic-labeled RGCs were collected using a magnetic separating unit.

### 2.4. Generation of Hypoxic Damage

The harvested RGCs were maintained for 24 h in a defined medium and then exposed to hypoxic conditions (0.3% O_2_) in a CO_2_ chamber_._ The level of hypoxic damage (0.3% O_2_) was controlled by varying the exposure time (2, 4, 6, 12, and 24 h). 

### 2.5. Bevacizumab Treatment

Primary RGCs were treated with different concentrations of bevacizumab (0.1, 0.4, 1.0, and 2.0 mg/mL), and RGCs were maintained in a defined medium for 24 h. Next, the RGCs were exposed to 4-h HPC and different concentrations of bevacizumab (0.4 mg/mL, 1.0 mg/mL, and 2.0 mg/mL) were added to the samples. Following 24 h of additional incubation in a defined medium, RGC survival rates were compared.

### 2.6. Cell Counting

Following HPC, the only insult thereafter was the natural death of RGCs over 24 h. Next, the RGCs were fixed with 4% paraformaldehyde for 30 min. After washing the RGCs in phosphate-buffered saline (PBS), a sufficient amount of DAPI stain solution was added to cover the RGCs. The cells were then incubated for 5 min and washed thrice in PBS. All steps were performed at room temperature. Five images of DAPI stained-RGCs were obtained for each sample using a light microscope (Olympus IX73, Olympus Corp., Tokyo, Japan) at 100× magnification. The number of RGCs in each image was manually counted and expressed as an average.

### 2.7. Flow Cytometry

Single-cell suspensions of RGCs were prepared according to a protocol described previously [13]. Briefly, following collagenase digestion, the isolated cells were stained with AnnexinV-FITC and propidium iodide (BioVision) and analyzed using FACS LSRII (Beckman Coulter, Brea, CA, USA).

### 2.8. Quantitative Reverse Transcription Polymerase Chain Reaction (RT-qPCR)

RNA was isolated with the RNeasy^®^ Micro Kit (Qiagen, Hilden, Germany) and reverse transcribed to cDNA using EcoDry^TM^ Premix (TaKaRa Bio, Mountain View, CA, USA). Real-time PCR was performed using SYBR^®^ Premix Ex Taq^TM^ (TaKaRa Bio) and preformulated primers. RT-qPCR was performed on the StepOnePlus™ Real-Time PCR System (Applied Biosystems/Thermo Fisher Scientific, Foster City, CA, USA). The list of primers used is presented in Table 1. The results were analyzed using the comparative cycle threshold (C_T_) method and normalized to those of *β-actin*, which was used as an internal control, in the same sample.

### 2.9. Enzyme-Linked Immunosorbent Assay (ELISA)

Commercially available ELISA kits against rat VEGF (RayBiotech Inc., Norcross, GA, USA) was used, and ELISA was performed according to the manufacturer’s instructions. Total protein concentrations were determined using the Pierce™ BCA Protein Assay Kit (Thermo Fisher Scientific, Inc., Rockford, IL, USA) as the standard. VEGF concentrations were measured by ELISA in triplicate. 

### 2.10. Western Blotting

Total cell lysates were obtained using a cell lysis buffer (Cell Signaling Technology) and incubated on ice for 5 min. The lysates were then sonicated, and the cell homogenates were centrifuged at 15,000× *g* for 15 min at 4 °C. Next, the concentration of proteins in the supernatants was measured using the Pierce™ Bicinchoninic Acid Protein Assay Kit (Thermo Fisher Scientific). Soluble proteins (30 μg per sample) were boiled for 5 min and resolved using 10% sodium dodecyl sulfate-polyacrylamide gel electrophoresis. Proteins were then electrotransferred to 0.45-μm-pore polyvinylidene fluoride membranes and blocked using 5% skim milk. Membranes were blotted overnight with primary antibodies diluted in 0.1% bovine serum albumin and 0.01% sodium azide in TBS-T against protein kinase B (Akt) (Cell Signaling Technology), focal adhesion kinase (FAK) (Cell Signaling Technology), and tubulin (Cell Signaling Technology). After washing three times with TBS-T, blots were incubated with horseradish peroxidase-conjugated secondary antibody (Cell Signaling Technology) for 1 h at room temperature. Blots were washed three times with TBS-T, and immunoreactive bands were visualized through enhanced chemiluminescence. Relative intensities of immunoreactive bands were measured after normalization for tubulin.

### 2.11. Statistics

All data are expressed as the mean ± standard deviation (SD). Differences between groups were examined using Student’s *t*-test and one-way analysis of variance was performed using SPSS Version 22.0 (IBM Corp., Armonk, NY, USA). A *p*-value < 0.05 was considered statistically significant.

## 3. Results

### 3.1. Outcome of Hypoxic Damage to RGCs Based on Exposure Time

**Figure 1:**
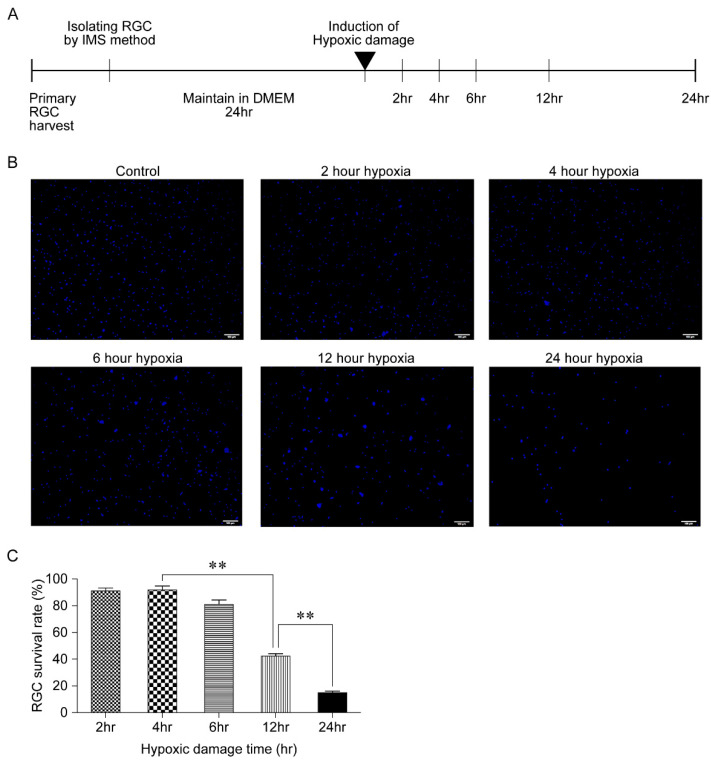
Consequence of varying durations of exposure to hypoxic conditions in primary retinal ganglion cells (RGCs). (**A**) After harvesting primary RGCs from the postnasal rat retinas, the cells were maintained for 24 h in a defined medium; then, hypoxic damage was inflicted on the cells in a CO_2_ chamber. After exposure to hypoxic conditions for 2, 4, 6, 12, and 24 h, the cells in each sample were counted. (**B**) Representative images of RGCs after exposure to hypoxic conditions for 2, 4, 6, 12, and 24 h. Scale bar, 100 µm. (**C**) Number of RGCs after exposure to hypoxic conditions for 2, 4, 6, 12, and 24 h. Data in the columns indicate the mean survival rate ± SD. Differences in RGC survival rates are indicated (** *p* < 0.01).

First, the response of RGCs exposed to hypoxic conditions for various lengths of time was measured to identify the sublethal HPC. Following treatment in DMEM/F-12 for 24 h, the harvested RGCs were exposed to hypoxic conditions by incubation in a CO_2_ chamber for 2, 4, 6, 12, and 24 h; next, the cells were counted (Figure 1A). As the RGCs were subjected to hypoxic damage, the cells began to aggregate (Figure 1B). In comparison to the non-hypoxia-damaged controls, the cell count ratio was 91.7%, 92.0%, 81.33%, 42.0%, and 14.7% after exposure to hypoxic conditions for 2, 4, 6, 12, and 24 h, respectively. There was no significant difference in the cell survival rate until 6 h of exposure to hypoxic conditions. However, after 12 h of exposure, the cell survival rate was significantly reduced (*p* < 0.001) (Figure 1C).

### 3.2. Effect of HPC on RGC Survival

**Figure 2:**
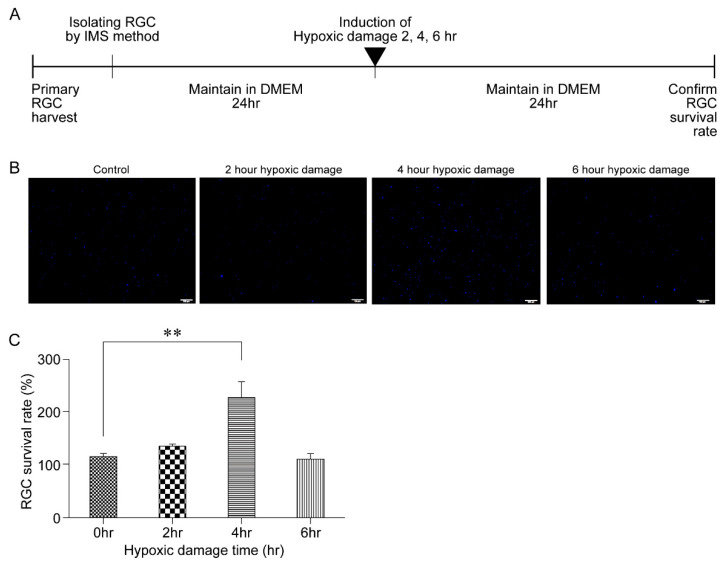
Effects of hypoxic preconditioning. (**A**) After inflicting hypoxic damage using the CO_2_ chamber, retinal ganglion cells (RGCs) were maintained for 24 h in a defined medium. After 24 h, the cell survival rate was compared. (**B**) Representative images of RGCs after exposure to hypoxic conditions for 2, 4, and 6 h. Scale bar, 100 µm. (**C**) The survival rate of RGCs after exposure to hypoxic conditions for 2, 4, and 6 h. Data in the columns indicate the mean survival rate ± SD. Differences in RGC survival rates are indicated (** *p* < 0.01).

To examine the effect of HPC on RGC survival, the cell survival rate between non-hypoxia-damaged cells and 2-, 4-, and 6-h hypoxia-damaged cells was compared. After inflicting hypoxic damage using the CO_2_ chamber for varying lengths of time, RGCs were additionally maintained for 24 h in a defined medium (Figure 2A). Qualitative comparison of hypoxia-damaged cells with that of non-hypoxia-damaged cells revealed a significant increase in cell survival in the 4-h hypoxia-damaged group (Figure 2B). The results of quantification of the number of cells for each group were in accordance with the observation: a significant increase in cell survival ratio was noted in the 4-h hypoxia-damaged group (*p* = 0.003) (Figure 2C).

### 3.3. HPC and VEGF

**Figure 3:**
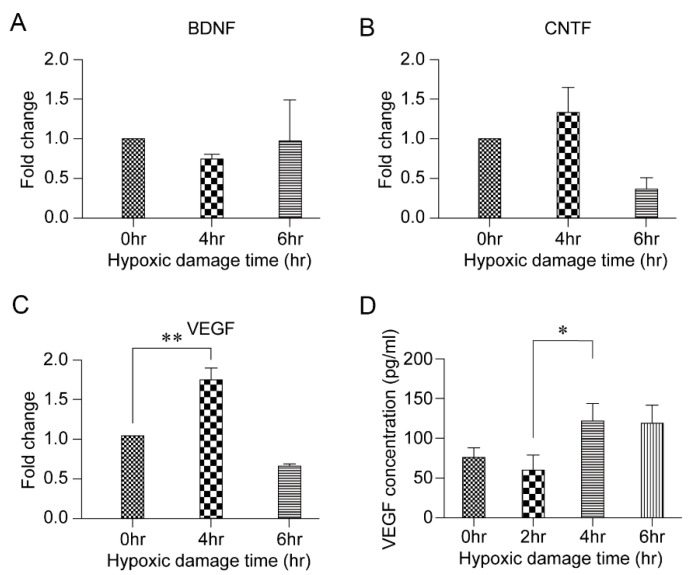
Vascular endothelial growth factor (VEGF) and hypoxic damage. (**A**–**C**). Fold changes in the expression of brain-derived neurotrophic factor (BDNF), ciliary neurotrophic factor (CNTF), and VEGF after hypoxic preconditioning of retinal ganglion cells (RGCs) for 4 and 6 h. Data in the columns indicate the mean fold changes ± SD. (**D**) VEGF concentration after hypoxic preconditioning of RGCs for varying lengths of time (2, 4, and 6 h). Data in the columns indicate the mean concentrations ± SD. Statistically significant differences in the mRNA fold change and VEGF concentration are indicated (* *p* < 0.05 and ** *p* < 0.01).

An in vitro model was established to determine the candidate factors that are associated with the protective role of HPC in RGC survival. The analysis of mRNA levels of VEGF, brain-derived neurotrophic factor (BDNF), and ciliary neurotrophic factor (CNTF) in the 4-h hypoxia-damaged RGCs revealed a significant increase in the VEGF mRNA levels (*p* = 0.006) (Figure 3A–C). This increase was confirmed upon analysis of the VEGF levels using ELISA (*p* = 0.043) (Figure 3D).

### 3.4. Effect of HPC and Anti-VEGF Antibody on RGC Survival

**Figure 4:**
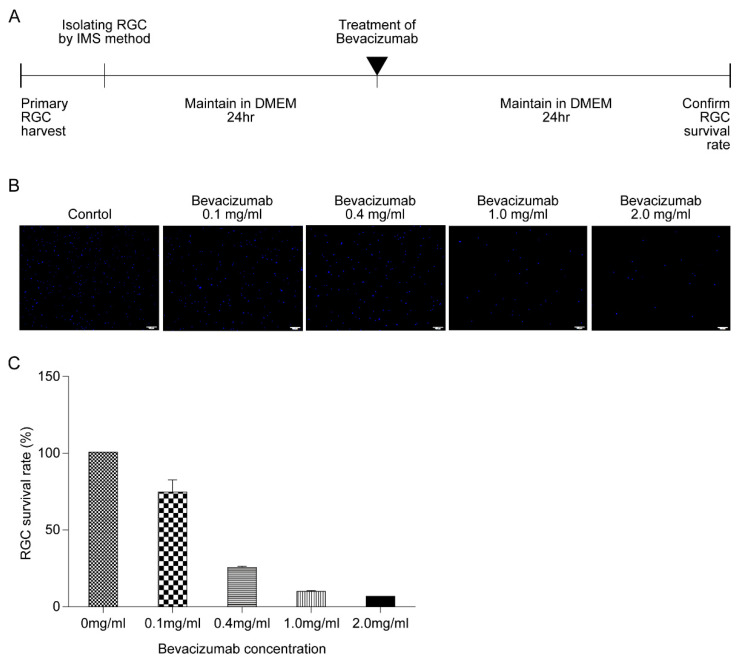
Retinal ganglion cell (RGC) survival rate according to antivascular endothelial growth factor (VEGF) antibody concentration. (**A**) After treatment with different concentrations of bevacizumab (0.1, 0.4, 1.0, and 2.0 mg/mL), RGCs were maintained in a defined medium for 24 h, and cell survival rates were compared. (**B**,**C**) Representative images and number of RGCs after bevacizumab treatment (0.1, 0.4, 1.0, and 2.0 mg/mL). Scale bar, 100 µm.

Next, to confirm the role of VEGF on HPC-induced RGC survival, the rate of RGC survival in response to bevacizumab (Avastin^®^) was investigated. First, the response of RGCs to varying concentrations of anti-VEGF antibody was verified by administering different concentrations of bevacizumab (0.1, 0.4, 1.0, and 2.0 mg/mL) and maintaining RGCs for 24 h in a defined medium (Figure 4A). Compared to the controls, the survival rate of RGCs decreased with increasing concentrations of anti-VEGF antibody in a concentration-dependent manner (Figure 4B,C).

**Figure 5:**
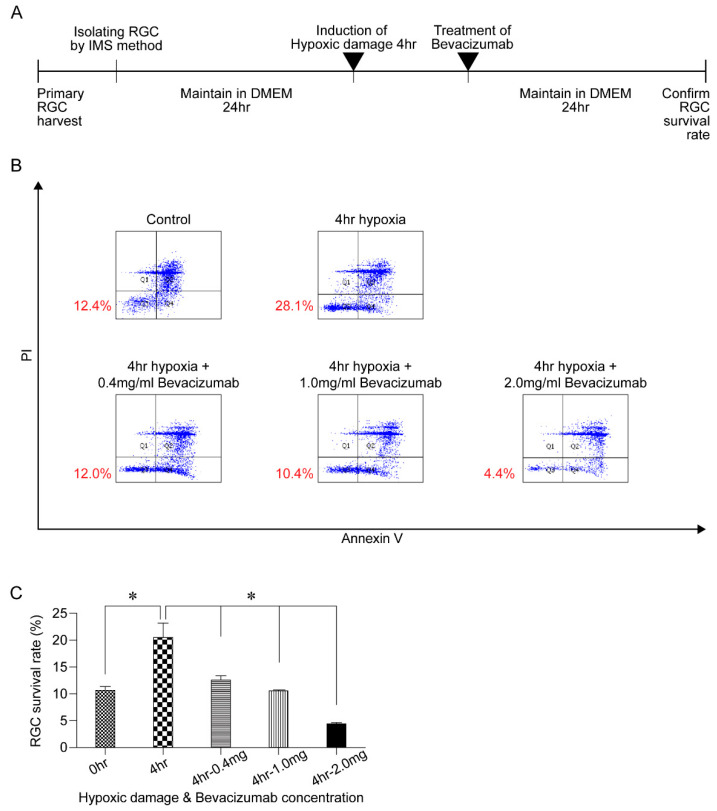
Effects of antivascular endothelial growth factor (VEGF) antibody on retinal ganglion cell (RGC) survival after hypoxic preconditioning (HPC). (**A**) After harvesting, primary RGCs were maintained for 24 h in a defined medium; then, the cells were subjected to HPC for 4 h in a CO_2_ chamber. Thereafter, different concentrations of bevacizumab (0.4, 1.0, and 2.0 mg/mL) were added to the samples. RGCs were maintained for another 24 h in a defined medium and cell survival was compared among the groups. (**B**,**C**) Using flow cytometry, the cells that survived HPC were verified and quantified. Data in the columns indicate the mean survival rate ± SD. Statistically significant difference from the 4-h HPC-treated cell group is indicated (* *p* < 0.05).

Next, the harvested RGCs were exposed to hypoxic conditions for 4 h and different concentrations of bevacizumab (0.4, 1.0, and 2.0 mg/mL) were added to the samples. Following 24 h of additional incubation in a defined medium, the cell survival rates were compared among treatment groups (Figure 5A). The flow cytometry results revealed that the survival rate significantly increased after 4 h of exposure to hypoxic conditions (*p* = 0.012). However, after adding bevacizumab, the cell survival rate decreased in a concentration-dependent manner (Figure 5B). Addition of 0.4 mg/mL and 1.0 mg/mL bevacizumab offset the positive effect of HPC. Moreover, the ratio of live cells decreased significantly following the addition of 2.0 mg/mL bevacizumab (*p* < 0.001) (Figure 5C).

### 3.5. VEGF Survival Pathway Following HPC

**Figure 6:**
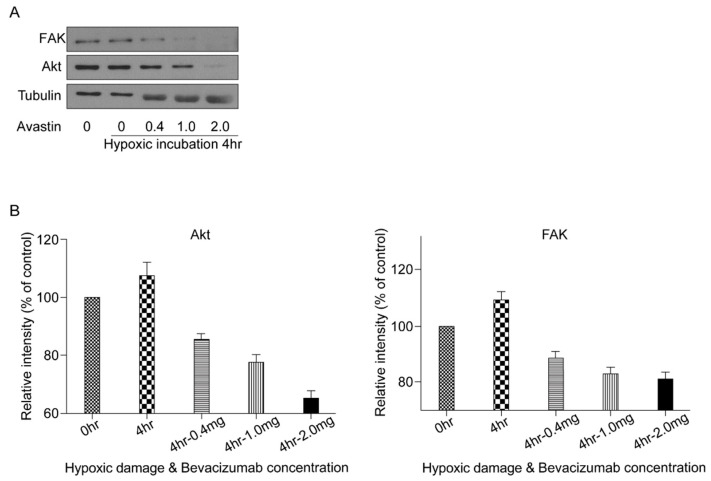
Hypoxic preconditioning and the vascular endothelial growth factor (VEGF) survival pathway. (**A**) Signaling molecules involved in pathways downstream of VEGF activation were investigated using immunoblot assays. Akt and focal adhesion kinase (FAK) levels were compared between the 4-h hypoxic preconditioning group and groups in which bevacizumab (0.4, 1.0, and 2.0 mg/mL) was administered. Representative gel images are shown. (**B**) Densitometry of Akt and FAK immunoblots. Data in the columns indicate the mean density ± SD, normalized to the level of tubulin in the same sample.

To investigate the underlying mechanism of action of VEGF in HPC-mediated RGC survival, signaling molecules known to be involved in prosurvival signaling pathways, such as Akt and FAK, were assessed under each treatment condition. Western blotting revealed an increase in Akt and FAK levels in cells subjected to HPC for 4 h. However, a stepwise decrease in Akt and FAK was elicited by treatment with higher concentrations of bevacizumab (Figure 6). 

## 4. Discussion

The present study examined the effect of HPC on RGC survival and the associated mechanism, including the role of HPC-induced VEGF. Firstly, the results showed that approximately 4 h of HPC significantly increased RGC survival compared to the control group. Second, HPC induced high levels of VEGF expression relative to other known survival factors. Third, the prosurvival effect of 4 h of HPC was offset by high dose anti-VEGF treatment. Finally, HPC-induced VEGF may promote the survival of RGCs by HPC through the Akt and FAK pathways. 

Hypoxia of the retina often occurs because of systemic conditions such as carotid artery stenosis, Takayasu’s arteritis, and hyperviscosity syndromes. Hypoxia may also occur due to retinal ischemia (characterized by retinal venous dilatation), retinal hemorrhages, retinal edema, and neovascularization [14]. Although the pathophysiology of hypoxic damage to the retina is not fully understood, it is generally accepted that hypoxia exerts a negative influence on RGC survival under various conditions including increased vascular permeability, disruption of the blood–retinal barrier, leakage of the fluid into the retinal tissue, and direct toxicity of the hypoxic damage [14,15,16,17].

In contrast, numerous previous studies have reported that under hypoxic conditions, the neural tissue is capable of triggering protective mechanisms within minutes and limiting cellular damage [5]. During a hypoxic episode, neuronal cells utilize adaptive mechanisms that allow them to survive and maintain homeostasis. The efficiency of energy-producing pathways is improved and that of energy-consuming processes, such as ion-motive ATPase and protein synthesis, is reduced. In addition, mitochondrial function is regulated to suppress apoptosis. HIF is also induced, and target genes of HIF such as erythropoietin and VEGF play a role in neuroprotection [18]. However, it has been reported that these protective mechanisms may disappear within hours, ultimately resulting in cell death [9]. The current findings support those of previous studies, which reported that increases in RGC death occur after more than 6 h of hypoxic conditioning and hypoxic conditions lasting less than 6 h increase RGC survival (Figure 1 and Figure 2).

Numerous studies have been conducted to identify molecules that promote RGC survival. Factors such as including nerve growth factor, BDNF, CNTF, VEGF, and insulin-like growth factors have been found [19,20,21,22]. Studies have also shown that the expression of several factors, including nitric oxide, inflammatory cytokines, glutamate, VEGF, and oxidative stress, is upregulated by hypoxia in the retina [8,23,24,25]. Consequently, the changes in VEGF, BDNF, and CNTF levels under HPC were investigated in this study. The results showed that VEGF expression was significantly upregulated in the hypoxic treatment group compared to that in the nonhypoxic control group. ELISA confirmed the increase (Figure 3). VEGF is a glycoprotein with a molecular weight of 46 kDa that binds to receptors on the surface of vascular endothelial cells to cause proliferation and increase capillary permeability [10,26]. Since it was first reported that hypoxia-induced VEGF may affect angiogenesis in 1992, [27] many studies have been conducted on the role of VEGF in retinal vasculature development as well as retinopathy of prematurity [28,29]. In recent studies, VEGF has been shown to promote the development and maturation of neural tissues, including the retina [8,30]. During development, VEGF is expressed by astrocytes in the RGC layer, cells of the inner nuclear layer, Müller cells, and retinal pigment epithelial cells [25,31]. In the adult retina, VEGF is expressed in the absence of active neovascularization and is implicated in the maintenance and function of adult retina neuronal cells [32]. Furthermore, VEGF exerts neuroprotective effects on injured RGCs in ocular hypertension animal models and delays their degeneration after axotomy [33,34,35]. In line with the previous studies, the current study also confirmed the protective role of VEGF in RGC survival following HPC. When VEGF activation was blocked with bevacizumab after 4 h of HPC, the RGC survival rate decreased in a dose-dependent manner (Figure 5). These results imply that the upregulation of VEGF expression occurs in many ischemic conditions of the retina.

Intravitreal injections of anti-VEGF antibodies, such as bevacizumab, are widely used in treatments to reduce angiogenesis based on studies that have reported VEGF expression upregulation in ischemic conditions such as AMD and diabetic retinopathy [10,11]. These high levels of VEGF can lead to retinal and vitreous hemorrhage, retinal detachment, and often blindness. However, according to the results in this study, repeated anti-VEGF antibody injections may have the potential risk of interfering with the neuroprotective action of VEGF. Similarly, a previous study demonstrated that small interfering RNA-mediated gene silencing of VEGF reduced the thickness of retinal cell layers, concluding that VEGF plays a neuroprotective role in the survival of the retinal neurons [32]. Although several studies claim the safety of anti-VEGF antibody treatment, [36,37,38] further studies are needed to thoroughly investigate any possible side effects of multiple anti-VEGF antibody injections for serious conditions of the eye such as glaucoma.

In an attempt to understand the signaling pathways downstream of VEGF, several candidate signaling molecules were studied. Western blot analyses revealed an increase in Akt and FAK levels in RGCs exposed to HPC for 4 h, which decreased upon treatment of the cells with anti-VEGF antibody (Figure 6). These results indicated that VEGF induces RGC survival following HPC primarily via the Akt and FAK pathways. VEGF has been shown to be a potent activator of the Akt pathway in other cell systems, including vascular endothelial cells [39]. Akt is activated by the phosphorylation of phosphoinositide 3-kinase (PI3K) and is involved in cell survival [34,40]. The FAK pathway is known to be involved in focal adhesion, cell migration, and cell survival [41,42]. In particular, VEGF activates FAK by increasing the affinity of integrin, and β1 integrin-FAK signaling has been found to modulate RGC survival [43,44]. The current study strongly suggests that endogenous retinal VEGF plays an important role via the Akt and FAK pathways in maintaining the viability of RGCs.

This study presents a number of aspects to consider in a clinical setting. First, excessive hypoxic damage exerts a negative impact on RGC survival. Second, HPC might contribute to enhanced RGC survival in stressful conditions such as glaucoma and diabetic retinopathy. In addition, bevacizumab may have unexpected harmful effects on RGC. A specific protocol for the treatment of neovascularization needs to be developed in the future.

This study has several limitations with respect to reproducing hypoxic conditions. First, the current study developed an in vitro model that focused on RGCs, despite the fact that in the body, RGCs are not isolated but coexist with various other cell types such as astrocytes, Müller cells, and other glial cells. Second, several factors other than VEGF may also be involved in cell survival following HPC, but not all of the correlations were analyzed. Third, the full extent of the VEGF survival pathway was not investigated. However, it seems that the Akt and FAK pathways may play a key role in RGC protection as reported previously. Finally, rat RGCs are not identical to human RGCs. 

## 5. Conclusions

HPC showed neuroprotective effects on primary RGC survival in this study. However, only specifically controlled exposure to hypoxic conditions rendered these neuroprotective effects. The in vitro model of this study showed that strong inhibition of VEGF countered HPC-mediated RGC survival, indicating that certain levels of HPC-induced VEGF may play an essential role in promoting cell survival during hypoxia.

## Figures and Tables

**Table 1:** Primers used for quantitative reverse transcription polymerase chain reaction.

Target Gene	Sequence
*BDNF*	F: 5′–GCG GCA GAT AAA AAG ACT GC–3′ R: 5′–GCC AGC CAA TTC TCT TTT TG–3′
*CNTF*	F: 5′–CAC CCC AAC TGA AGG TGA CT–3′ R: 5′–ACC TTC AAG CCC CAT AGC TT–3′
*VEGF*	F: 5′–GGC TCT GAA ACC ATG AAC TTT CT–3′ R: 5′–GCA GTA GCT GCG CTG GTA GAC–3′
*Akt*	F: 5′–GTG GCA AGA TGT GTA TGA G–3′ R: 5′–CTG GCT GAG TAG GAG AAC–3′
*FAK*	F: 5′–AAA ATG TGA CGG GCC TAG TG–3′ R: 5′–TAC TCC TGC TGA AGG CTG GT–3′
*β-Actin*	F: 5′–CAC CCG CGA GTA CAA CCT T–3′ R: 5′–CCC ATA CCC ACC ATC ACA CC–3′

BDNF: Brain-derived neurotrophic factor; CNTF: Ciliary neurotrophic factor; VEGF: Vascular endothelial growth factor; Akt: Protein kinase B (PKB); FAK: focal adhesion kinase; F, forward; R, reverse.

## Data Availability

No new data were created or analyzed in this study. Data sharing is not applicable to this article.
